# Measurement of anatomical parameters of anterior transpedicular root screw intervertebral fusion system of cervical spine

**DOI:** 10.1186/s12891-023-06995-6

**Published:** 2023-11-21

**Authors:** Sen-qi Ye, Liu-jun Zhao, Zhi-peng Hou, Ji-hui Zhang, Liang Yu, Yong-jie Gu

**Affiliations:** 1 Department of Spinal Surgery, Yuyao People’s Hospital, Ningbo, 315499 Zhejiang Province China; 2 Department of Spinal Surgery, Ningbo No.6 hospital of Ningbo University, Ningbo, Zhejiang Province 315040 China; 3 Health Science Center, Ningbo University, Ningbo, 315211 Zhejiang Province China

**Keywords:** Cervical spine, Anterior approach, Pedicle screw, Intervertebral fusion system

## Abstract

**Objective:**

This study aims to investigate the feasibility of the anterior transpedicular root screw (ATPRS) intervertebral fusion system for the cervical spine and provide a basis for the design of the ATPRS intervertebral fusion system.

**Methods:**

A total of 60 healthy adult cervical spine CT images examined from our hospital were selected, including 30 males and 30 females, with an average age of 39.6 ± 4.8 years. The image data was imported into Mimics 21.0 software in DICOM format for 3D model reconstruction. Simulated screw insertion was performed on both sides of the midline of the intervertebral space. The entry point (P_1_) was determined when the upper and lower screw paths did not overlap. When the screw was tangent to the medial edge of the Luschka joint, the insertion point was determined as the entry point (P_2_). Measurements were taken and recorded for the following parameters: distance from the screw entry point to the midline of the intervertebral space (DPM), the simulated screw length, inclination angle, cranial/caudal tilted angle, the anterior–posterior (AP) and mediolateral (ML) diameters of the cervical intervertebral space, the heights of the anterior, middle, and posterior edges of the cervical intervertebral space, and the curvature diameter of the lower end plate of the cervical vertebral body. Statistical analysis was performed on the measurement results.

**Results:**

The screw entry area (P_1_P_2_) showed an increasing trend from C3-C7 in both male (2.92–6.08 mm) and female (2.32–5.12 mm) groups. There were statistical differences between men and women at the same level (*P* < 0.05). The average screw length of men and women was greater than 20 mm, and the upper and lower screw lengths showed an increasing trend from C3 to C7. In the area where screws could be inserted, the range of screw inclination was as follows: male group upper screw (47.73–66.76°), lower screw (48.05–65.35°); female group upper screw (49.15–65.66°) and lower screw (49.42–63.29°); The range of cranial/caudal tilted angle of the screw was as follows: male group upper screw (32.06–39.56°), lower screw (29.12–36.95°); female group upper screw (30.97–38.92°) and lower screw (27.29–37.20°). The anterior–posterior diameter and mediolateral diameter of the cervical intervertebral space showed an increasing trend from C3 to C7 in both male and female groups. The middle height (MH) of the cervical intervertebral space was greater than the anterior edge height (AH) and posterior edge height (PD), with statistical differences (*P* < 0.05).

**Conclusions:**

Through the study of CT images of the cervical spine, it was determined that the ATPRS intervertebral fusion system has a feasible area for screw insertion in the cervical intervertebral space.

## Introduction

The anterior transpedicular screw (ATPS) technology was proposed in 2008 by Koller et al. [1] they subsequently conducted experimental studies demonstrating that the pullout resistance of ATPS was 2.5 times higher than that of VBS (vertebral body screws) [2]. Furthermore, they provided evidence supporting the comparable stability of ATPS to combined anterior and posterior fixation [3]. The results of Wu et al. [4] also illustrated the excellent biomechanical properties of ATPS, especially in the osteoporotic group. Because of its excellent biomechanical properties, ATPS has attracted many scholars' research [5–7]. And so far, there have been several reports on the clinical application of ATPS. For example, Zhang et al. [8] reported a successful case of ATPS fixation in a patient with cervical spine tuberculosis, and the good effect was maintained at the 2-year follow-up examination.

**Fig. 1:**
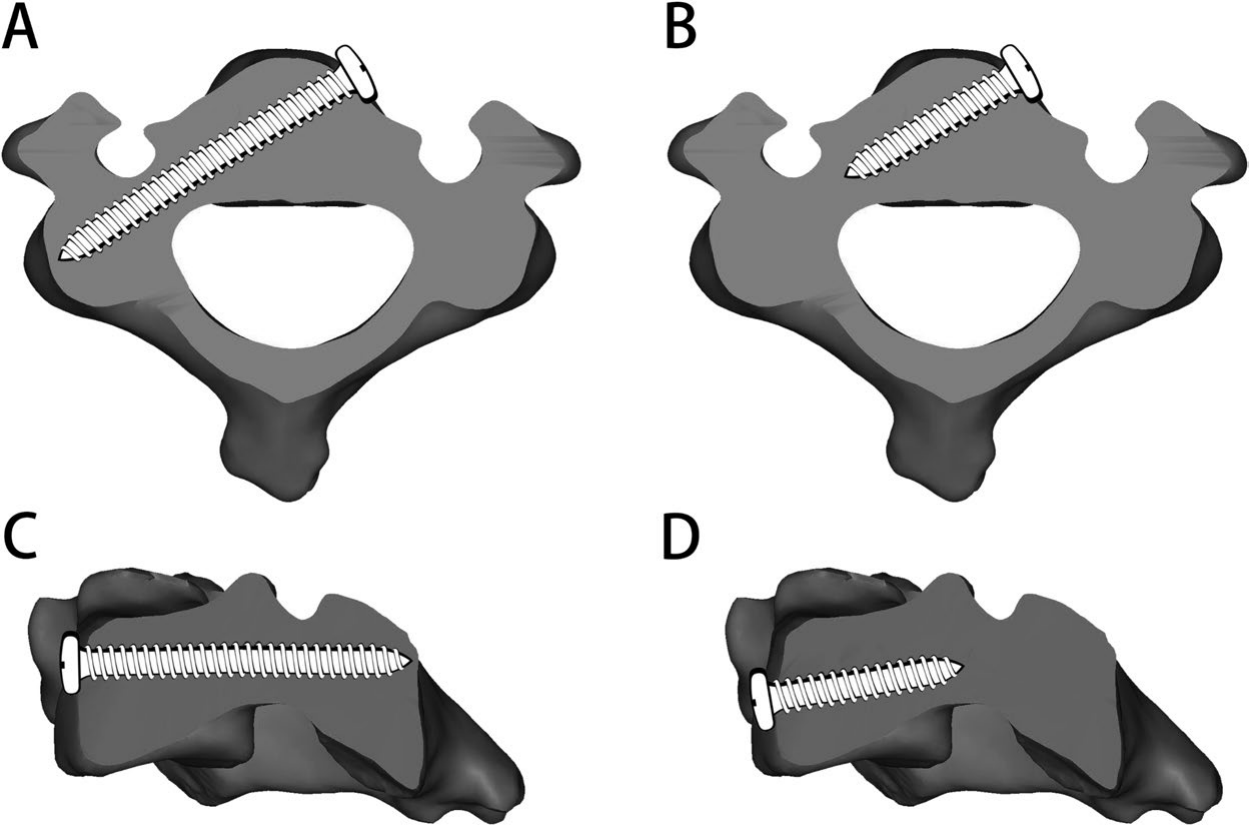
Traditional ATPS technology (**A**, **C**) and improved ATPRS technology (**B**, **D**)

**Fig. 2:**
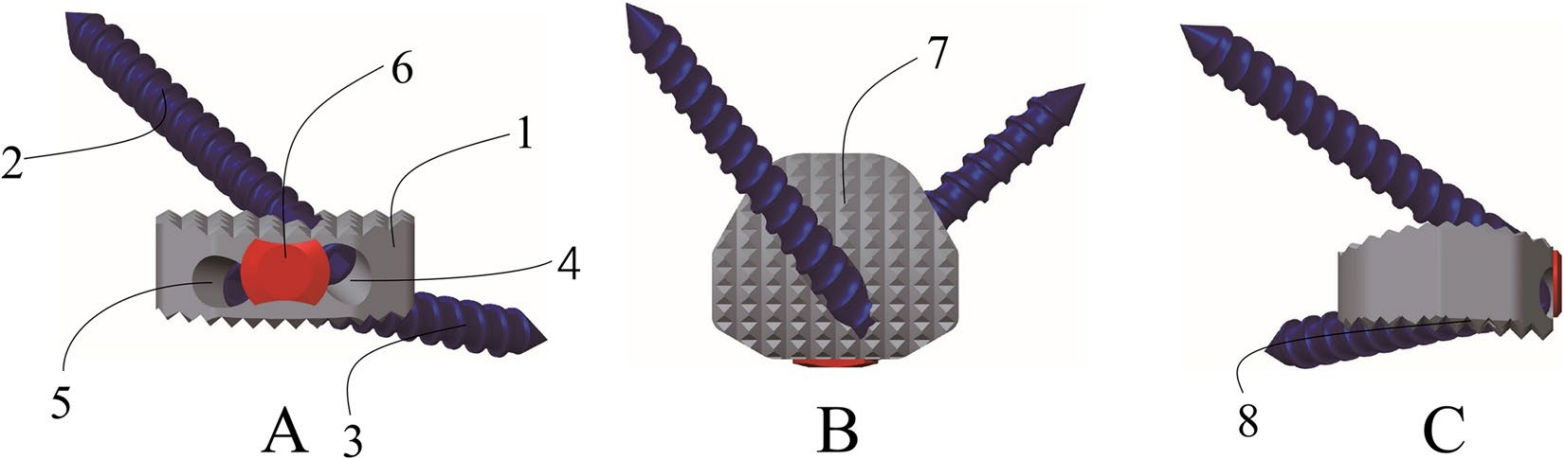
Schematic diagram of ATPRS intervertebral fusion system: front view (**A**), horizontal view (**B**), sagittal view (**C**), the ATPRS intervertebral fusion system includes an insertion (1), first screw (2), second screw (3), first tapered hole (4), second tapered hole (5), locking cap (6), upper surface (7), lower surface (8)

However, based on previous research, our team found some limitations of ATPS technology in clinical applications: 1. the upper screw entry point of ATPS is too close to the upper edge of the vertebral body, and the distance between the plate and the upper intervertebral disc is too close. This proximity may accelerate degeneration of the upper segment intervertebral disc. 2. The ATPS necessitates complete penetration of the pedicle during screw insertion, requiring high levels of accuracy. This increases the risk of injury to the vertebral artery, nerve root, and cervical spinal cord [5, 9, 10]. Considering these shortcomings, our team innovatively proposed the anterior transpedicular root screw (ATPRS) technology [11] (Fig. 1). This approach involves inserting the screw head at the root of the pedicle, eliminating the need for complete pedicle penetration. Theoretically, this enhances the safety and operability of screw insertion. Previous study has shown that ATPRS technology has a larger range of screw insertion on the vertebral body compared with ATPS technology [11]. Besides, another study indicated that there were no statistical differences (*P* > 0.05) in the CT values of the bone surrounding the ATPRS path compared to those of the ATPS. However, the CT values were found to be statistically different (*P* < 0.05) when compared to the VBS [12]. These results suggested that the ATPRS technique can contact a concentrated area of the cortical bone in the vertebral body, thereby enhancing the screw's stability. However, the plates and screws of the anterior cervical screw plate system protrude from the cervical vertebral body, resulting in direct contact and friction with the soft tissue of the anterior cervical region. This can lead to postoperative dysphagia, anterior cervical foreign body sensation, esophageal injury, etc. [13]. Additionally, multi-level titanium plate fusion and fixation have been associated with an increased incidence of degenerative lesions in adjacent segments [14–16]. The zero-profile (Zero-P) fusion device can solve the above problems of the anterior cervical screw plate fixation system, effectively reduce the incidence of postoperative dysphagia, and slow down the degeneration of adjacent segments [17, 18]. Also, the Zero-P can still play a similar clinical effect as the traditional anterior plate decompression and internal fixation system in the treatment of multi-segmental cervical spondylotic myelopathy, and reduce the incidence of postoperative dysphagia [19]. Therefore, it is envisaged to combine the Zero-P fusion device and ATPRS technology to design the ATPRS intervertebral fusion system. This system consists of three main parts: the insert positioned between the upper and the lower vertebral bodies, the first screw connecting the upper vertebral body and the insert, and the second screw connecting the lower vertebral body and the insert (Fig. 2). This study aims to explore the feasibility ATPRS intervertebral fusion system, and provide data support for its design.

## Materials and methods

### Patient data

In our research, a total of 60 healthy patients, who underwent cervical CT scans at our institution from May 2021 to August 2021 were retrospectively selected, including 30 males and 30 females, aged 25–50 years, with a mean age of 39.6 ± 4.8 years. In addition, all patients were excluded from any infectious, traumatic, neoplastic, degenerative disease, congenital, or developmental involving the spine. All patients were scanned by a helical CT scanner (Philips Brilliance 64-channel scanner, Netherlands). The study protocol was approved by the institutional ethics committee of Ningbo No.6 Hospital of Ningbo University (CODE: k2020019) and written informed consent from all study participants.

### Model reconstruction

**Fig. 3:**
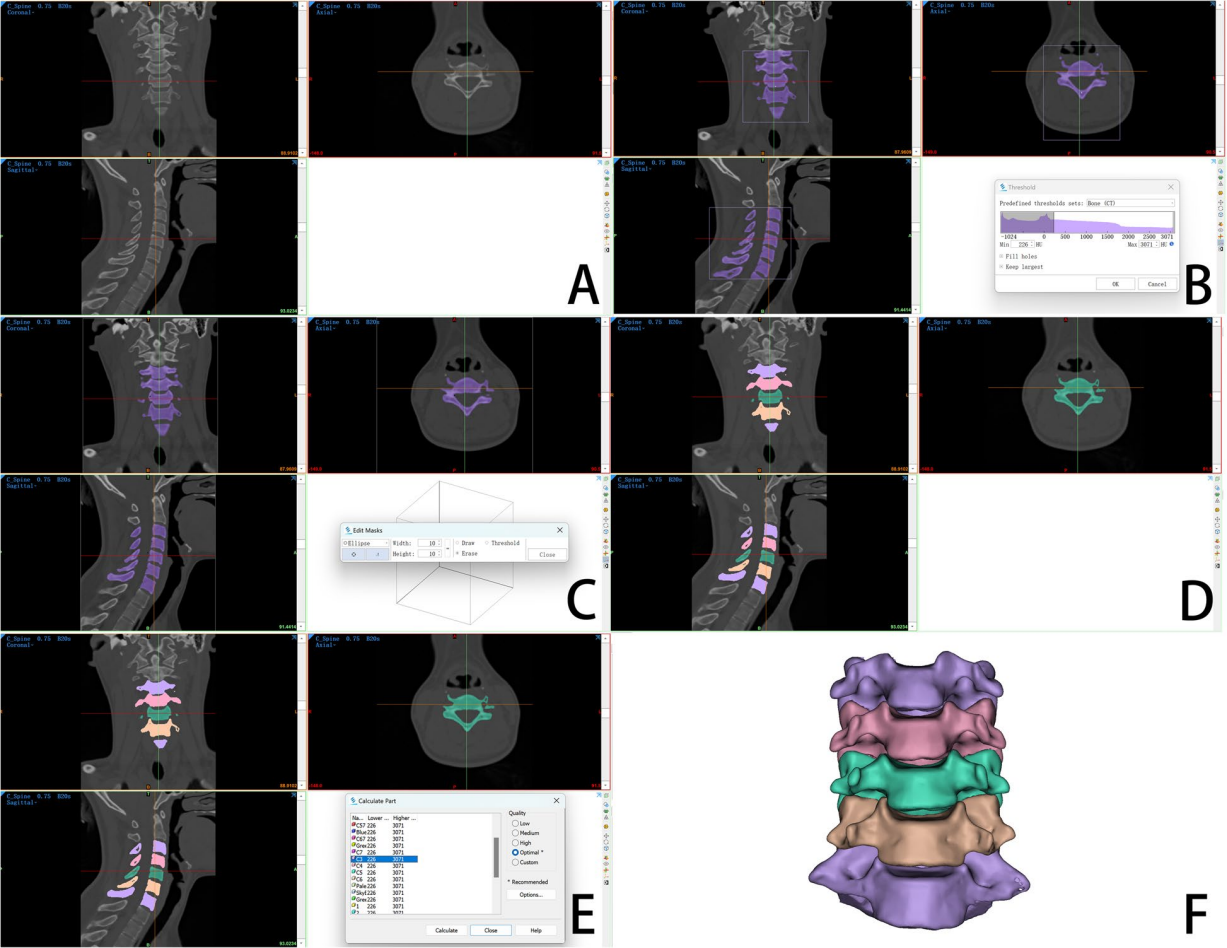
3D model reconstruction of cervical spine: import cervical spine CT image data (**A**), bone threshold selection (**B**), mask editing (**C**), vertebral body segmentation (**D**), model 3D reconstruction calculation (**E**), 3D models of cervical spine (**F**)

All the CT images were imported into Mimics Medical 21.0 (Materialise, Belgium) in DICOM format for three-dimensional reconstruction of the cervical spine model (Fig. 3). The specific steps were as follows: (1) threshold selection: in this study, the normal bone tissue threshold range (226-3071HU) was used to select the required bone structure. (2) Editing mask: we used the region growth function to extract the cervical vertebral bone structure and remove other unconnected bone structures and used the editing mask tool to manually edit and process each layer of the cervical spine CT image. (3) Vertebral body segmentation: we segmented the lower cervical vertebrae C3-C7 and gave different colors to distinguish them. (4) Three-dimensional calculation: the three-dimensional model of the cervical spine was reconstructed by performing three-dimensional reconstruction calculation on the processed cervical mask. (5) Optimization: the three-dimensional cervical model was further optimized. Finally, the high-quality and high-precision three-dimensional image model of cervical C3-C7 was obtained.

### Simulated screw insertion

**Fig. 4:**
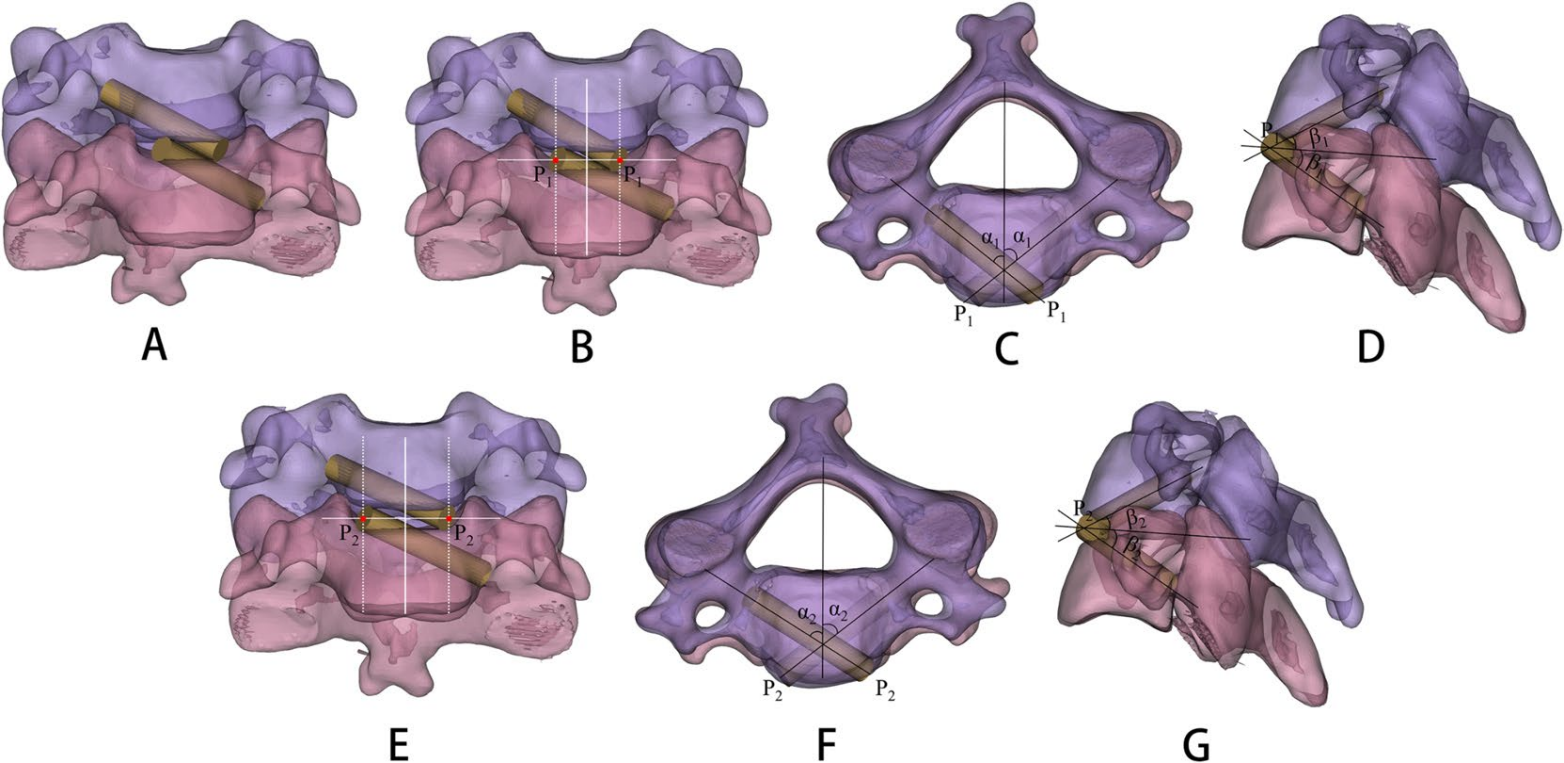
The simulation of screw insertion in Mimics software: the upper and lower screw paths do not overlap (**A**), the screw entry point (P_1_) nearest to the midline of the intervertebral space (**B**), the angle α_1_ formed between the simulated screw axis and mid-sagittal plane (**C**), the angle β_1_ formed between the middle section of the cervical intervertebral space and the simulated screw axis (**D**); the screw entry point (P_2_) farthest from the midline of the intervertebral space (**E**), the angle α_2_ formed between the simulated screw axis and mid-sagittal plane (**F**), the angle β_2_ formed between the middle section of the cervical intervertebral space and the simulated screw axis (**G**)

In the analysis module of Mimics Medical 21.0, a 3.5 mm diameter cylinder was created as a screw to simulate screw insertion. The transparency of the vertebral body was adjusted to medium. Then simulated screw insertion was performed on both sides of the midline of the intervertebral space (the line connecting the midpoint of the anterior wall of the lower edge of the upper vertebral body and the midpoint of the upper edge of the anterior wall of the lower vertebral body). And the simulated screw head was positioned at the intersection of the posterolateral edge of the vertebral body and the axis of the pedicle in the horizontal plane, as well as close to the lower edge of the pedicle in the sagittal plane [11]. In the process of screw insertion, where the upper screw path and the lower screw path did not overlap, was defined as the nearest screw insertion point (P_1_) of the ATPRS intervertebral fusion system to the midline of the intervertebral space (Fig. 4). Previous studies have shown that the maximum width of the interbody fusion cage should not exceed the transverse diameter of the intervertebral space, that is, the line connecting the medial edge of the Luschka joint [20]. Therefore, when the screw of the ATPRS intervertebral fusion system was tangent to the medial edge of the Luschka joint, the screw entry point at this time was determined to be the farthest screw insertion point from the middle line of the intervertebral space (P_2_) (Fig. 4). Considering that the cervical vertebral body is not completely symmetrical, when the screw was tangent to the medial edge of the Luschka joint on any side, it can be considered as tangent.

### Parameter measurement and significance

To minimize errors, through sagittal, horizontal, and coronal fluoroscopy, screws were precisely inserted into anatomical positions. Meanwhile, all parameters were measured three times by a senior spine surgeon, and the average was used as the final value [21, 22].

### Measurement of intervertebral space related parameters

**Fig. 5:**
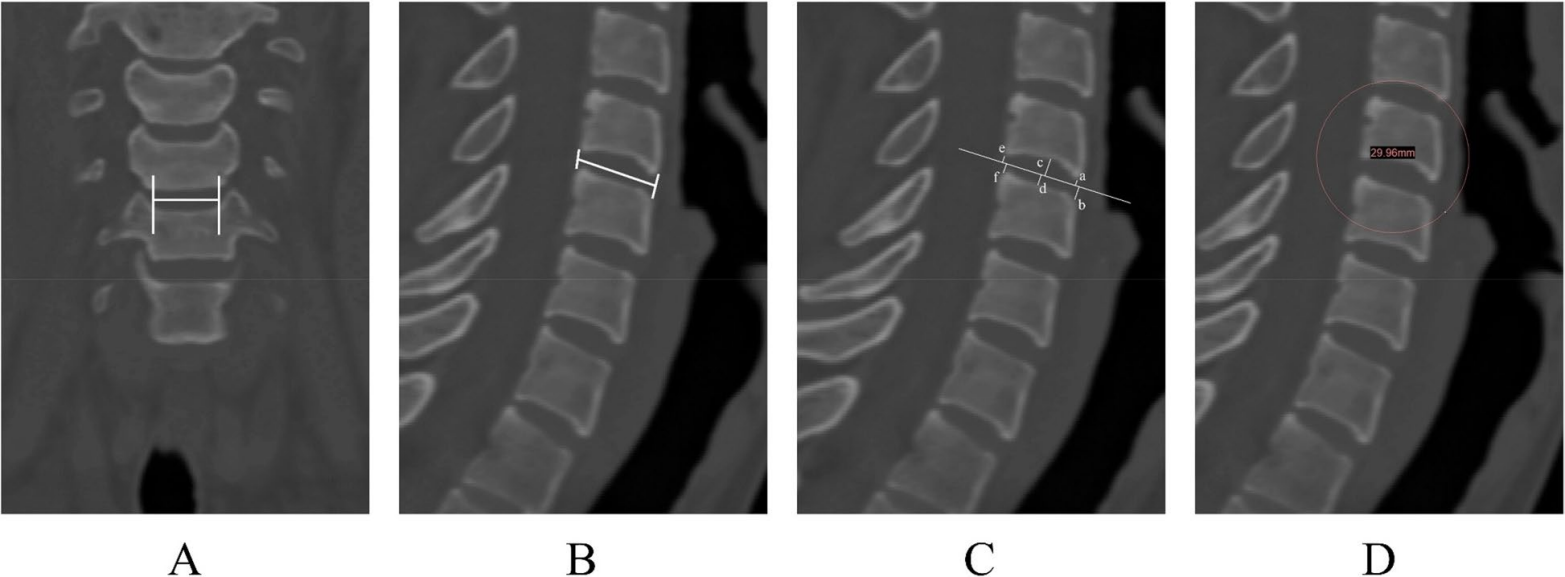
Measurement of intervertebral space related parameters: measurement of ML diameter of the cervical intervertebral space (**A**), measurement of AP diameter of the cervical intervertebral space (**B**), measurement of AH, MH, and PH of the cervical intervertebral space (**C**), measurement of curvature diameter of the lower endplate of the cervical vertebra (**D**)

The relevant parameters of the intervertebral spaces were measured. (1) Mediolateral (ML) diameter of the intervertebral space: that was to measure the distance of the line connecting the medial edge of the Luschka joint as a reference for the design of the left and right width of the ATPRS intervertebral fusion system (Fig. 5A). (2) Anterior–posterior (AP) diameter of the intervertebral space: that was to measure the distance from the midpoint of the line between the anterior lower edge of the upper vertebral body and the anterior upper edge of the lower vertebral body to the midpoint of the line between the posterior lower edge of the upper vertebral body and the posterior upper edge of the lower vertebral body, as a reference for the design of the anterior and posterior length of the ATPRS intervertebral fusion system (Fig. 5B). (3) Anterior edge height (AH) of intervertebral space: that was to measure the distance from the anterior lower edge of the upper vertebral body to the middle section of intervertebral space + the distance from the anterior upper edge of the lower vertebral body to the middle section of intervertebral space (a + b) (Fig. 5C). (4) Middle height (MH) of intervertebral space: that was to measure the distance from the highest point of the lower endplate of the upper vertebral body to the middle section of intervertebral space + the distance from the lowest point of the upper endplate of the lower vertebral body to the middle section of intervertebral space (c + d) (Fig. 5C). (5) Posterior edge height (PH) of intervertebral space: that was to measure the distance from the posterior lower edge of the upper vertebral body to the middle section of intervertebral space + the distance from the posterior upper edge of the lower vertebral body to the middle section of intervertebral space (e + f) (Fig. 5C). The AH, MH and DH of the intervertebral space can be used as a reference for the design of the height of the ATPRS intervertebral fusion system. (6) Curvature diameter of the lower endplate: that was to measure the curvature diameter of the lower endplate of the upper vertebral body (Fig. 5D). This parameter can be used as a reference for the design of curvature changes of ATPRS interbody fusion system so that the upper surface of the fusion system insert can fit the lower end plate of the upper vertebral body as much as possible.

### Measurement of relevant parameters of screw insertion

The simulated screw was inserted into the vertebral body at point P_1_, measured and recorded the relevant parameters (Fig. 4). (1) Distance from point P_1_ to the midline of intervertebral space (DMP_1_); (2) Simulated screw length (SL_1_); (3) α_1_ angle: the angle between the simulated screw axis and the midsagittal plane of the vertebral body on the horizontal plane; (4) β_1_ angle: the angle between the simulated screw axis and the middle section of the intervertebral space in the sagittal plane. The simulated screw was inserted into the vertebral body at point P_2_, measured and recorded the relevant parameters (Fig. 4). (1) Distance from P_2_ to the middle line of intervertebral space (DMP_2_); (2) Simulated screw length (SL_2_); (3)α_2_ angle: the angle between the simulated screw axis and the midsagittal plane of the vertebral body on the horizontal plane; (4)β_2_ Angle: the angle between the simulated screw axis and the middle section of the intervertebral space in the sagittal plane. Then DMP_2_—DMP_1_ was the screw entry area (P_1_P_2_). The above parameters can be used as a reference for the design of the screw entry position, screw length, and screw entry direction of the ATPRS intervertebral fusion system.

### Statistical analysis

Data were described as mean and standard deviation (Mean ± SD). The independent samples t-tests were performed to compare the data between left and right on the same cervical level. If the variances of the data were homogeneous, ANOVA was used to compare the data of C3-C7; if they were not, a non-parametric test was used. *P* < 0.05 was considered statistically significant. All analyses were performed using SPSS 21.0 software (SPSS, Chicago, Illinois, USA). The results of statistical analysis were plotted using GraphPad Prism 9.5 software (* = *p* < 0.05, ** = *p* < 0.01, *** = *p* < 0.001, **** = *p* < 0.0001).

## Results

A total of 60 cases of cervical spine CT imaging data were observed and measured in this study, including 30 males and 30 females. A total of 5040 data were summarized as follows statistical analysis.

### The transverse and sagittal diameters of the cervical intervertebral space

**Fig. 6:**
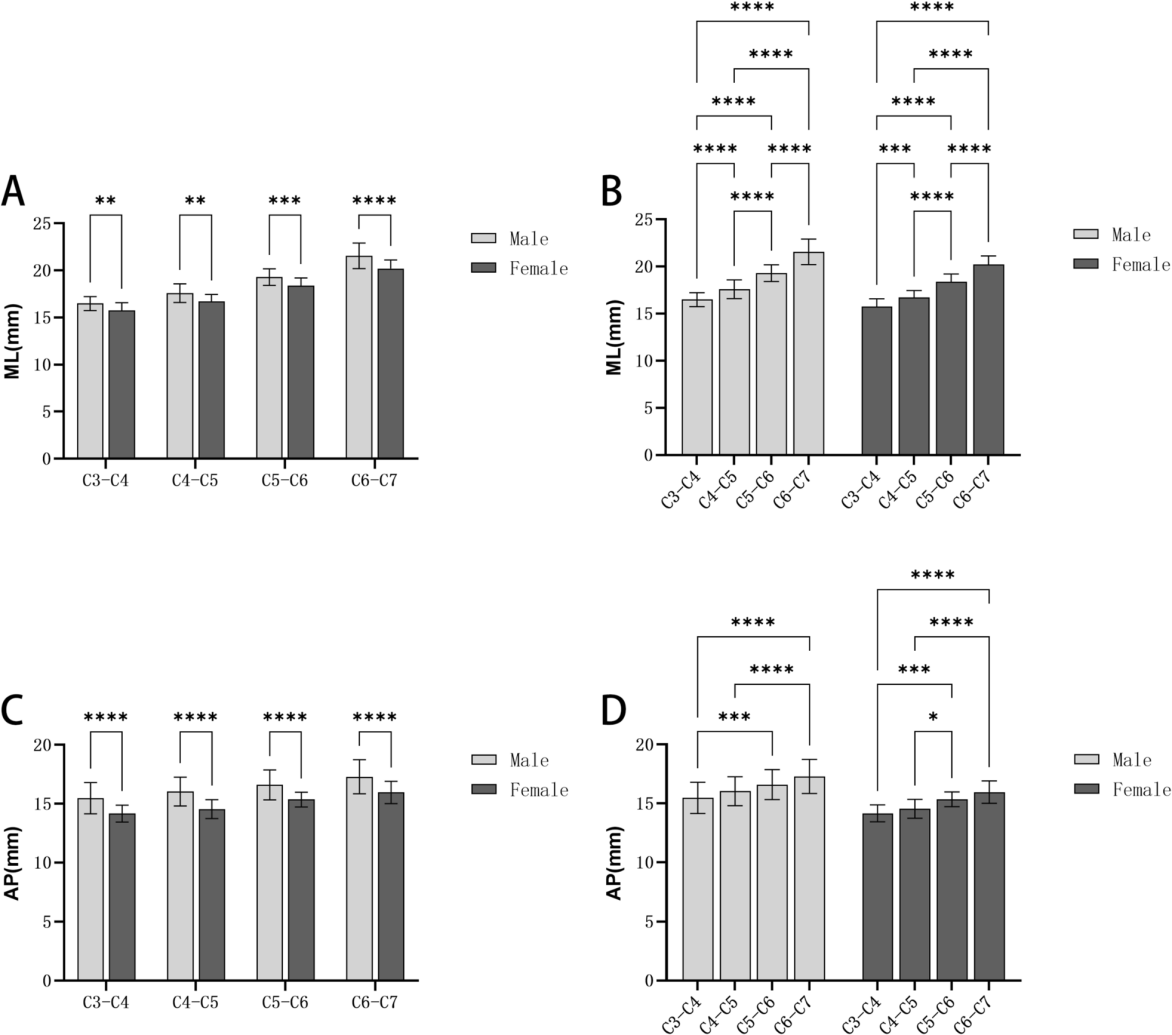
Comparison of intervertebral space diameters: **A**: Comparisons between men and women of the ML diameter; **B**: Comparisons between different segments of the ML; **C**: Comparisons between men and women of the AP; **D**: Comparisons between different segments of the AP

Table 1 illustrates that the average ML diameter range of C3-C7 intervertebral space was (16.48–21.55) mm in the male group, (15.75–20.20) mm in the female group; From C3 to C7, the mean AP diameter range was (15.47–17.29) mm in the male group, (14.16–15.95) mm in the female group. The ML diameter and AP diameter of cervical intervertebral space in both male and female groups showed an increasing trend from C3 to C7. Moreover, the average value of ML and AP diameters of cervical intervertebral space in the male group were greater than those in the female group within the same segment, and these differences were statistically significant (*p* < 0.05) (Fig. 6).

**Table 1:** ML diameter and AP diameter of cervical intervertebral spaces ($\overline{x }$±s, mm)

Level	ML		AP	
	Male	Female	Male	Female
C3-C4	16.48 ± 0.74	15.75 ± 0.82	15.47 ± 1.33	14.16 ± 0.71
C4-C5	17.59 ± 0.98	16.72 ± 0.73	16.04 ± 1.23	14.53 ± 0.80
C5-C6	19.29 ± 0.88	18.37 ± 0.83	16.59 ± 1.27	15.35 ± 0.62
C6-C7	21.55 ± 1.35	20.20 ± 0.90	17.29 ± 1.45	15.95 ± 0.95

### The heights of the anterior, middle, and posterior edges of the cervical intervertebral space

**Fig. 7:**
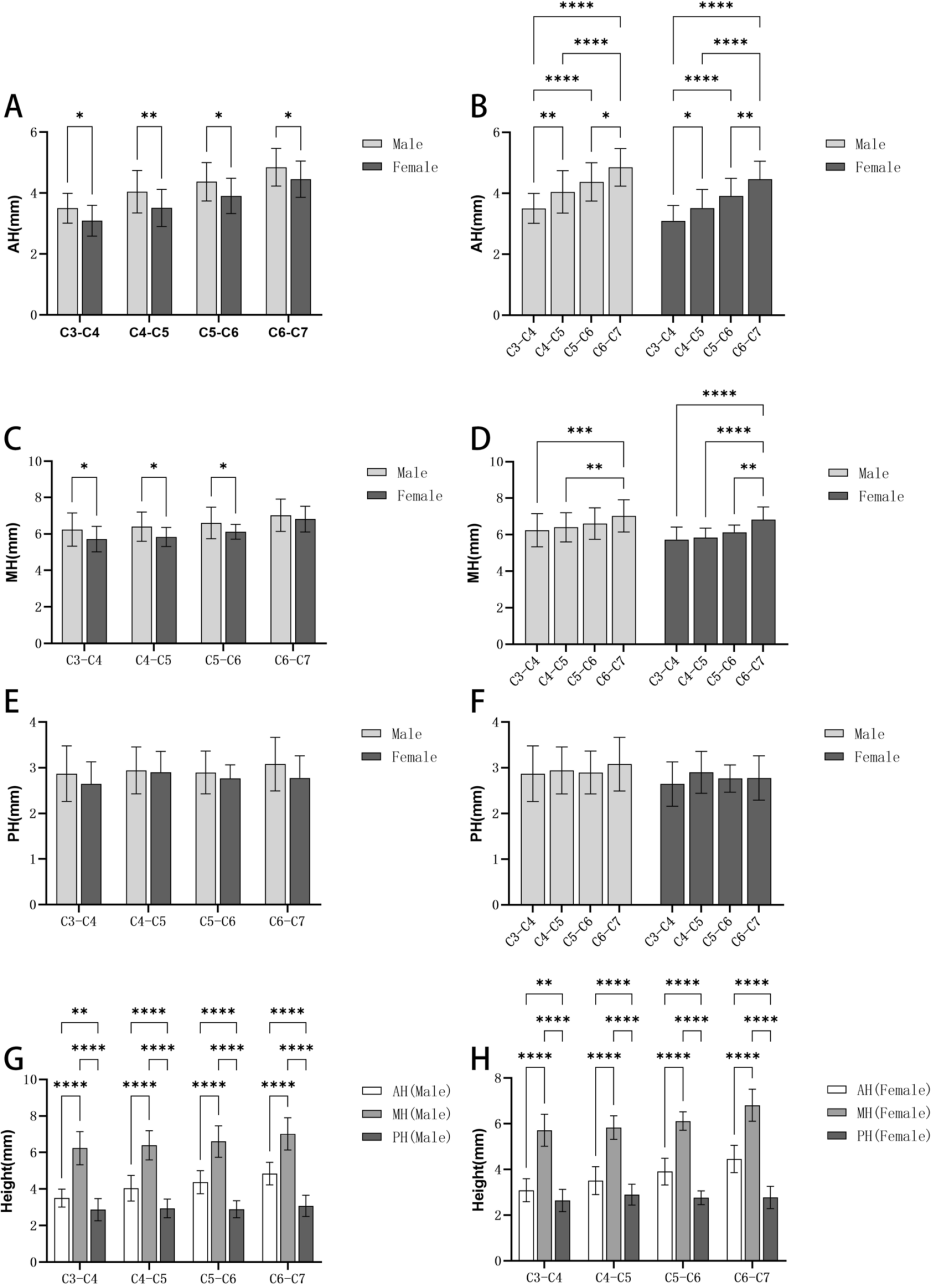
Comparison of intervertebral space height: **A**: Comparisons between men and women of the AH; **B**: Comparisons between different segments of the AH; **C**: Comparisons between men and women of the MH; **D**: Comparisons between different segments of the MH; **E**: Comparisons between men and women of the PH; **F**: Comparisons between different segments of the PH; **G**: Height comparison of the AH, MH, and PH in males; **H**: Height comparison of the AH, MH, and PH in females

In Table 2, the average AH of the C3-C7 intervertebral space in the male group was (3.50–4.85) mm, the average MH was (6.24–7.02) mm, and the average PH was (2.87–3.08) mm; In the female group, the average AH of C3-C7 intervertebral space was (3.09–4.46) mm, the average MH was (5.72–6.81) mm, and the average PH was (2.64–2.90) mm. The AH and MH of the male and female groups increased from C3 to C7, and the AH and MH of the cervical intervertebral space at the same segment in the male group were greater than those in the female group (Fig. 9). Overall, in the male group or the female group, the height of the cervical intervertebral space at the same segment showed the MH > AH > PH, and there were statistical differences (*p* < 0.05) (Fig. 7).

**Table 2:** AH, MH, and PH of the cervical intervertebral space ($\overline{x }$±s, mm)

Level	AH		MH		PH	
	Male	Female	Male	Female	Male	Female
C3-C4	3.50 ± 0.49	3.09 ± 0.69	6.24 ± 0.91	5.72 ± 0.70	2.87 ± 0.61	2.64 ± 0.49
C4-C5	4.04 ± 0.70	3.51 ± 0.61	6.39 ± 0.80	5.83 ± 0.52	2.94 ± 0.51	2.90 ± 0.70
C5-C6	4.37 ± 0.63	3.91 ± 0.58	6.60 ± 0.86	6.12 ± 0.40	2.90 ± 0.47	2.76 ± 0.30
C6-C7	4.85 ± 0.62	4.46 ± 0.60	7.02 ± 0.88	6.81 ± 0.70	3.08 ± 0.59	2.78 ± 0.49

### The curvature diameter of the lower end plate of the cervical vertebral body

**Fig. 8:**
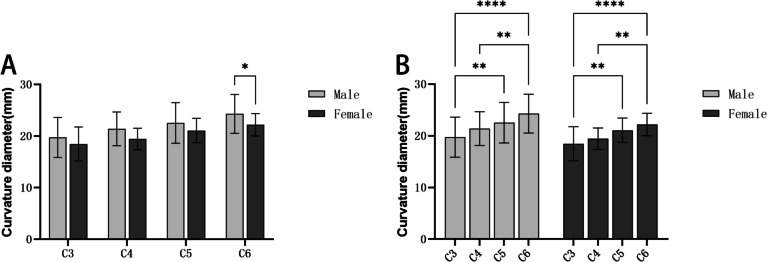
Comparison of curvature diameter of lower endplate: **A**: Comparisons between men and women of the curvature diameter; **B**: Comparisons between different segments of the curvature diameter

In Table 3, the average curvature diameter range of the lower endplate of C3-C6 was (19.74–24.30) mm in the male group, (18.46–22.19) mm in the female group. The curvature diameter of the lower cervical endplate in both male and female groups showed an increasing trend from C3 to C6. Additionally, the curvature diameter of the lower cervical endplate in the male group was greater than that in the female group at the same segment, but only in C6, there was a statistical difference between men and women (*P* < 0.05) (Fig. 8).

**Table 3:** The curvature diameter of the lower endplate of the cervical vertebra ($\overline{x }$±s, mm)

Lower endplate	Curvature diameter	
	Male	Female
C3	19.74 ± 3.87	18.46 ± 3.30
C4	21.40 ± 3.26	19.44 ± 2.08
C5	22.53 ± 3.93	21.07 ± 2.36
C6	24.30 ± 3.76	22.19 ± 2.18

### The distance from the screw entry point to the midline of the intervertebral space and the screw entry area

**Fig. 9:**
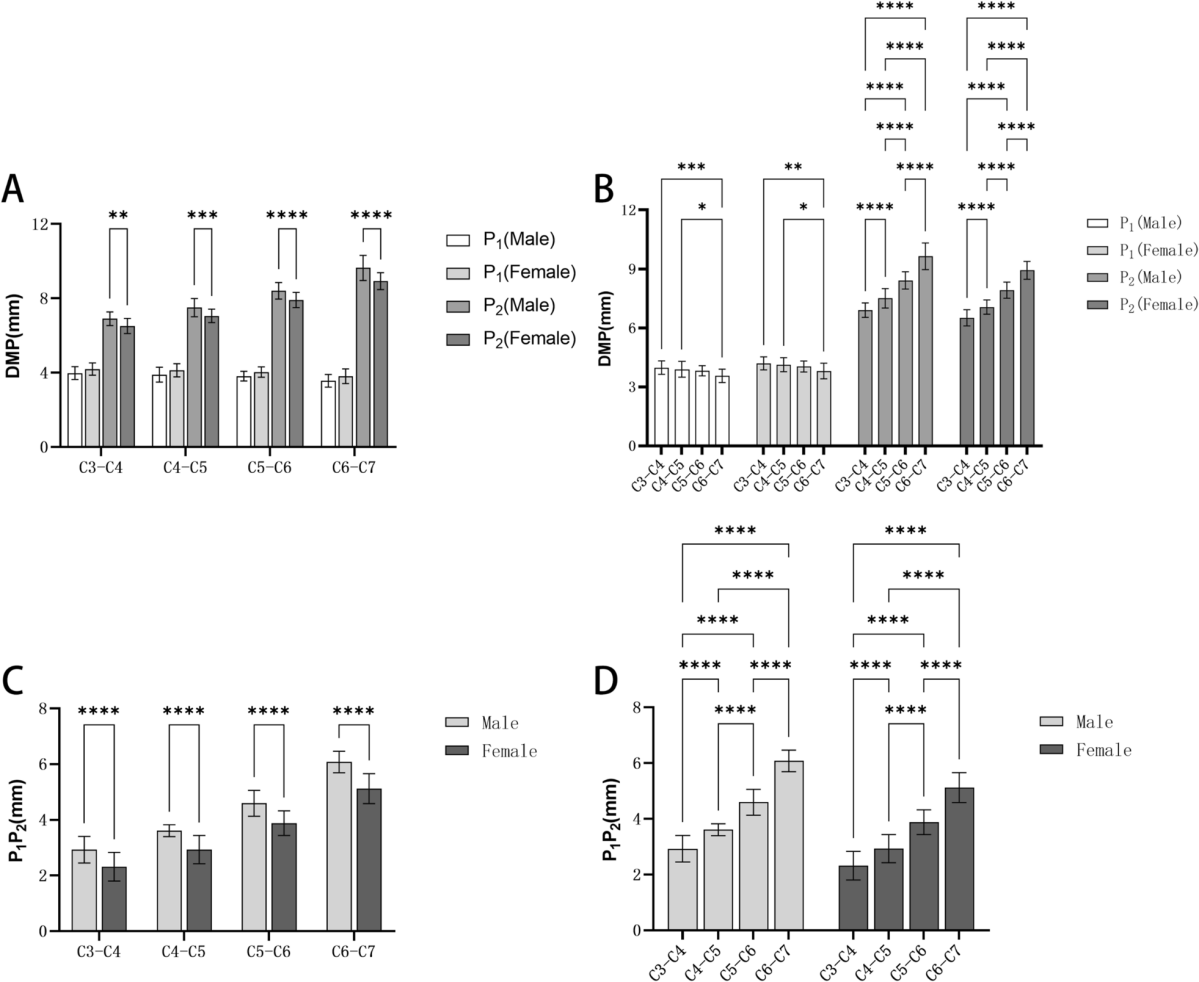
Comparison of the distance between the entry points to the midline of the intervertebral space: **A**: Comparisons between men and women; **B**: Comparisons between different segments; **C**: Comparisons between men and women of the screw insertion area; **D**: Comparisons between different segments of the distance between the screw insertion area

It can be seen from Table 4 that the average DMP_1_ of the C3-C7 range was (3.56–3.98) mm in the male group, (3.81–4.20) mm in the female group; the average DMP_2_ of the C3-C7 range was (6.90–9.64) mm in the male group, (6.51–8.93) mm in the female group. The average DMP_1_ in both male and female groups showed a decreasing trend from C3 to C7. Besides, the average DMP_1_ in the same segment in the male group was smaller than that in the female group, but there were no significant differences (*P* > 0.05) (Fig. 9). Conversely, the average DMP_2_ showed an increasing trend from C3-C7 in both the male and female groups. Moreover, the average DMP_2_ in the same segment in the male group was greater than that in the female group, and there were statistical differences (*P* < 0.05) (Fig. 9).

**Table 4:** The distance between the entry points to the midline of the intervertebral space and the distance between the entry points ($\overline{x }$±s, mm)

Level	DMP_1_		DMP_2_		P_1_P_2_	
	Male	Female	Male	Female	Male	Female
C3-C4	3.98 ± 0.34	4.20 ± 0.33	6.90 ± 0.37	6.51 ± 0.41	2.92 ± 0.48	2.32 ± 0.51
C4-C5	3.89 ± 0.40	4.13 ± 0.36	7.50 ± 0.49	7.05 ± 0.36	3.61 ± 0.21	2.93 ± 0.51
C5-C6	3.82 ± 0.26	4.03 ± 0.28	8.41 ± 0.44	7.92 ± 0.41	4.59 ± 0.46	3.88 ± 0.44
C6-C7	3.56 ± 0.34	3.81 ± 0.40	9.64 ± 0.68	8.93 ± 0.46	6.08 ± 0.39	5.12 ± 0.53

The screw entry area (P_1_P_2_), that is DMP_2_—DMP_1_, ranged from (2.92–6.08) mm in the male group and (2.32–5.12) mm in the female group. And the average value of P_1_P_2_ in both male and female groups showed an increasing trend from C3 to C7. In the same segment, the average value of P_1_P_2_ in the male group was greater than that in the female group, and there were statistical differences (*P* < 0.05). Additionally, there were statistically significant differences in the P_1_P_2_ of cervical intervertebral space at different segments (*P* < 0.05) (Fig. 9).

### The simulated screw length

**Fig. 10:**
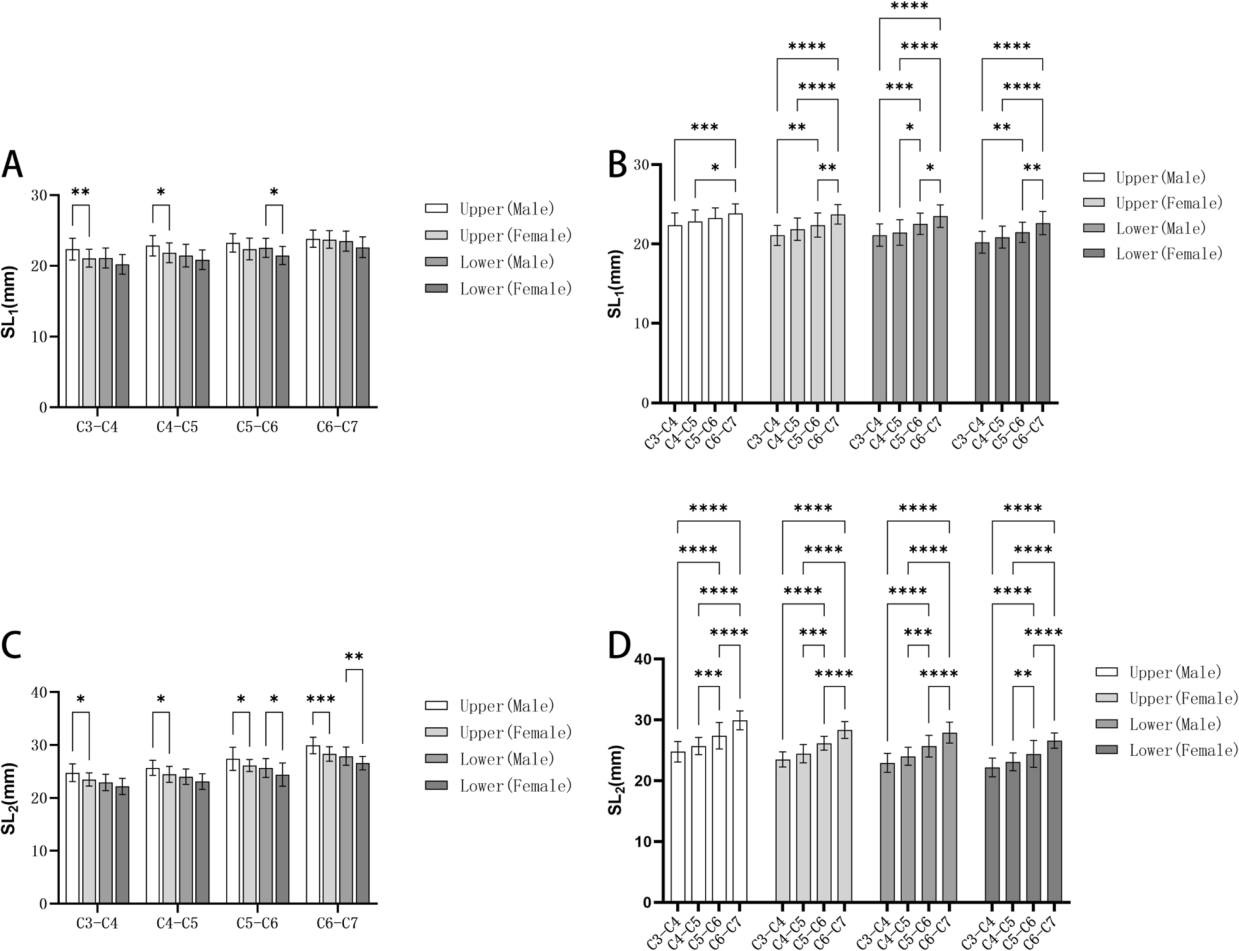
Comparison of screw length: **A**: Comparisons between men and women of SL_1_; **B**: Comparisons between different segments of SL_1_; **C**: Comparisons between men and women of SL_2_; D: Comparisons between different segments of SL_2_

In Table 5, the average SL_1_ of the upper screw of the C3-C7 range was (22.35–23.68) mm in the male group, and (21.07–22.94) mm in the female group; the average SL_1_ of the lower screw of the C3-C7 range was (21.10–23.49) mm in the male group, and (20.20–22.62) mm in the female group. And in Table 6, the average SL_2_ of the upper screw of the C3-C7 range was (24.56–29.61) mm in the male group, and (23.50–28.32) mm in the female group; the average SL_2_ of the lower screw of the C3-C7 range was (22.74–27.5) mm in the male group, and (22.18–26.59) mm in the female group. No matter at P_1_ or P_2_ point, the length of the upper and lower screws in the male or female group showed an increasing trend from C3 to C7, and the average length of the upper or lower screws in the male group was greater than that in the female group at the same segment of the intervertebral space. The comparison between males and females in the same segment of SL_1_ and SL_2_, as well as the comparison between different segments within the same gender, are illustrated in Fig. 10.

**Table 5:** SL_1_ at P_1_ point in the cervical intervertebral space ($\overline{x }$±s, mm)

Level	Upper screw		Lower screw	
	Male	Female	Male	Female
C3-C4	22.35 ± 1.53	21.07 ± 1.26	21.10 ± 1.41	20.20 ± 1.38
C4-C5	22.83 ± 1.45	21.84 ± 1.40	21.43 ± 1.60	20.85 ± 1.38
C5-C6	23.23 ± 1.30	22.37 ± 1.53	22.53 ± 1.35	21.45 ± 1.28
C6-C7	23.68 ± 1.23	22.94 ± 1.46	23.49 ± 1.42	22.62 ± 1.46

**Table 6:** SL_2_ at P_2_ point in the cervical intervertebral space ($\overline{x }$±s, mm)

Level	Upper screw		Lower screw	
	Male	Female	Male	Female
C3-C4	24.56 ± 1.69	23.50 ± 1.24	22.74 ± 1.54	22.18 ± 1.53
C4-C5	25.50 ± 1.41	24.46 ± 1.51	23.82 ± 1.47	23.11 ± 1.48
C5-C6	27.00 ± 2.17	26.55 ± 1.13	25.47 ± 1.78	24.41 ± 2.20
C6-C7	29.61 ± 1.56	28.32 ± 1.38	27.50 ± 1.72	26.59 ± 1.25

### The screw angles

**Fig. 11:**
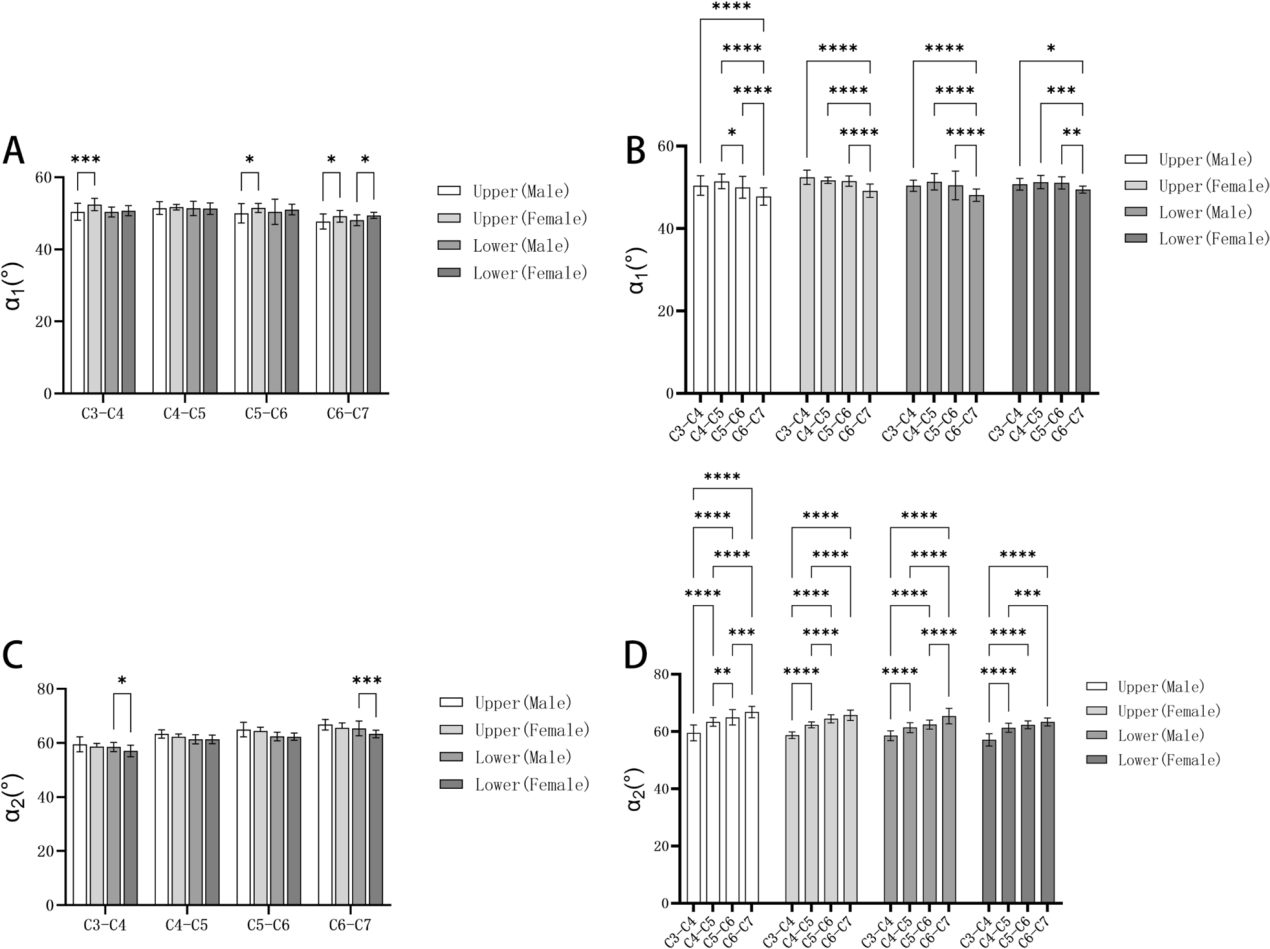
Comparison of α: **A**: Comparisons between men and women of the angle α_1_; **B**: Comparisons between different segments of the angle α_1_; **C**: Comparisons between men and women of the angle α_2_; **D**: Comparisons between different segments of the angle α_2_

From Table 7, the average α_1_ of upper screw form C3 to C7 was (47.73–51.44) ° in the male group and (49.15–52.41) ° in the female group; the average α_1_ of lower screw form C3 to C7 was (48.05–51.36) ° in male group and (49.42–51.27) ° in the female group. In Table 8, the average α_2_ of upper screw form C3 to C7 was (59.46–66.76) ° in male group and (58.67–65.66) ° in female group; the average α_2_ of lower screw form C3 to C7 was (58.49–65.35) ° in male group and (57.04–63.29) ° in female group. Furthermore, in both male and female groups, statistical differences existed between the upper and lower screws α_1_ angle and upper and lower screws α_2_ angle at the same segment (*P* < 0.05). The comparison between men and women in the same segment of the screw α_1_ angle or α_2_ angle and the comparison between different segments of the same sex are shown in Fig. 11.

**Table 7:** The angle α_1_ in the cervical intervertebral space ($\overline{x }$±s, °)

Level	Upper screw		Lower screw	
	Male	Female	Male	Female
C3-C4	50.43 ± 2.37	52.41 ± 1.70	50.35 ± 1.35	50.73 ± 1.40
C4-C5	51.44 ± 1.78	51.68 ± 0.78	51.36 ± 1.99	51.27 ± 1.62
C5-C6	49.99 ± 2.68	51.48 ± 1.24	50.43 ± 3.51	51.05 ± 1.48
C6-C7	47.73 ± 2.11	49.15 ± 1.62	48.05 ± 1.50	49.42 ± 0.85

**Table 8:** The angle α_2_ in the cervical intervertebral space ($\overline{x }$±s, °)

Level	Upper screw		Lower screw	
	Male	Female	Male	Female
C3-C4	59.46 ± 2.76	58.67 ± 1.15	58.49 ± 1.71	57.04 ± 2.14
C4-C5	63.32 ± 1.54	62.28 ± 1.05	61.33 ± 1.73	61.29 ± 1.59
C5-C6	64.91 ± 2.69	64.40 ± 1.41	62.37 ± 1.59	62.31 ± 1.36
C6-C7	66.76 ± 1.96	65.66 ± 1.81	65.35 ± 2.73	63.29 ± 1.40

**Fig. 12:**
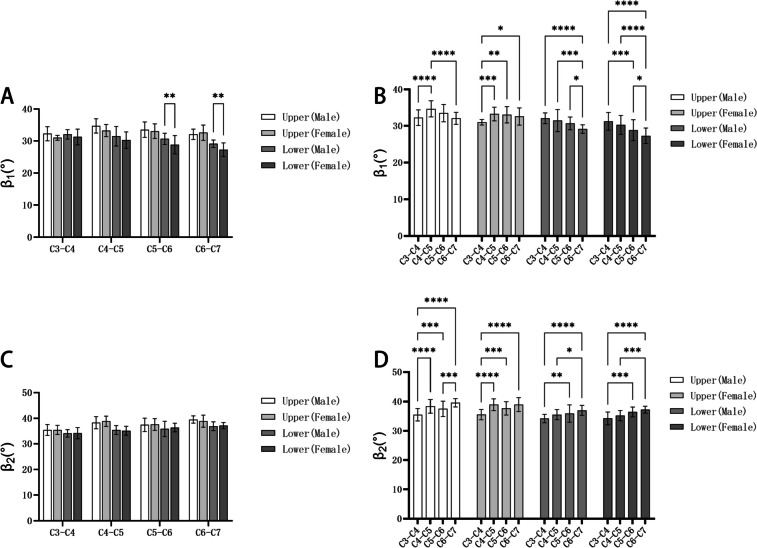
Comparison of β: **A**: Comparisons between men and women of the angle β_1_; **B**: Comparisons between different segments of the angle β_1_; **C**: Comparisons between men and women of the angle β_2_; **D**: Comparisons between different segments of the angle β_2_

From Table 9, the average β_1_ of upper screw form C3 to C7 was (32.06–34.67) ° in the male group and (30.97–33.25) ° in the female group; the average β_1_ of lower screw form C3 to C7 was (29.12–32.04) ° in male group and (27.29–31.24) ° in the female group. In Table 10, the average β_2_ of upper screw form C3 to C7 was (35.47–39.56) ° in the male group and (35.50–38.92) ° in the female group; the average β_2_ of lower screw form C3 to C7 was (34.18–36.95) ° in male group and (34.23–37.20) ° in the female group. Furthermore, in both male and female groups, statistical differences existed between the upper and lower screws β_1_ angle and upper and lower screws β_2_ angle at the same segment (*P* < 0.05). Figure 12 illustrates the comparison between males and females in the same segment for both the β_1_ and β_2_ angles of the screw, as well as the comparison between different segments within the same gender.

**Table 9:** The angle β_1_ in the cervical intervertebral space ($\overline{x }$±s, °)

Level	Upper screw		Lower screw	
	Male	Female	Male	Female
C3-C4	32.25 ± 2.19	30.97 ± 0.76	32.04 ± 1.47	31.24 ± 2.24
C4-C5	34.67 ± 2.24	33.25 ± 1.90	31.46 ± 3.04	30.24 ± 2.57
C5-C6	33.49 ± 2.41	33.05 ± 2.28	30.66 ± 1.74	28.82 ± 2.84
C6-C7	32.06 ± 1.64	32.58 ± 2.38	29.12 ± 1.16	27.29 ± 2.11

**Table 10:** The angle β_2_ in the cervical intervertebral space ($\overline{x }$±s, °)

Level	Upper screw		Lower screw	
	Male	Female	Male	Female
C3-C4	35.47 ± 2.12	35.50 ± 1.75	34.18 ± 1.43	34.23 ± 2.16
C4-C5	38.34 ± 2.34	38.90 ± 2.01	35.49 ± 1.71	35.18 ± 1.75
C5-C6	37.49 ± 2.62	37.64 ± 2.28	35.90 ± 2.95	36.43 ± 1.65
C6-C7	39.56 ± 1.42	38.92 ± 2.37	36.95 ± 1.69	37.20 ± 1.91

## Discussion

Anterior cervical decompression and fusion (ACDF), as a standard surgical procedure for the treatment of cervical degenerative and traumatic diseases, is widely used in clinics [23]. This approach is preferred because it directly lessens spinal cord compression and offers a better biomechanical condition for fusion and is less intrusive than posterior surgery. However, the conventional anterior approach for more than three segments or cervical severe three-column injuries is a challenge. Previous literature has also indicated that the use of a single cortical vertebral screw in anterior surgery is not enough to reconstruct the stability after multi-level vertebral resection, and it was prone to loosening failure of internal fixation after surgery [24, 25]. Wang et al. [26] believed that assisted posterior fixation can increase the stability of anterior fixation and reduce the incidence of surgical failure and complications. But combined anterior and posterior surgery not only prolongs the operation time and causes greater trauma, but also increases the economic burden of patients [27]. The proposal of ATPS technology provides an alternative solution for such patients, but its shortcomings limit its application in clinical practice. In addition, the anterior cervical plate screw system increases the risk of dysphagia, anterior cervical foreign body sensation, and esophageal injury [14–16]. Multilevel titanium plate fusion fixation also increases the incidence of adjacent vertebral diseases. To address these issues, it was envisaged to combine the ATPRS technology with Zero-P to design an internal fixation system. This system can provide sufficient stability while reducing postoperative swallowing difficulties, the sensation of a foreign body in the neck, and the occurrence of adjacent vertebral diseases. This study aims to measure relevant parameters of the ATPRS interbody fusion system through cervical spine CT images.

Mimics software, as a professional interactive medical image control system, can convert two-dimensional images into three-dimensional models, which can more intuitively measure and observe the corresponding tissues and organs. It has been widely used in orthopedics [28, 29]. In this study, Mimics software was used to measure the related parameters of the ATPRS intervertebral fusion system in imaging, thereby circumventing measurement errors arising from the physiological curvature of the cervical spine and discrepancies in patient positioning encountered with traditional imaging measurement tools.

By measuring the CT images of the cervical spine, it was found that the ML diameter of C3-C7 intervertebral space was (16.48–21.55 mm), and the AP diameter of C3-C7 intervertebral space was (15.47–17.29 mm) in the male group. The ML diameter of the C3-C7 intervertebral space was (15.75–20.20 mm), and the AP diameter of the C3-C7 intervertebral space was (14.16–15.95 mm) in the female group. This is compared with the C3-C7 intervertebral space ML diameter range (16.13 ± 1.99 mm) and intervertebral space AP diameter range (16.08 ± 1.84 mm) measured by Wang et al. [30]. The intervertebral space ML diameter in this study was larger, but the intervertebral space AP diameter has little difference. Besides, the increasing trend of transverse diameter and sagittal diameter of cervical intervertebral space from C3 to C7 was consistent. Dong et al. [31] measured the intervertebral discs of C4-C7 segments of 138 healthy Chinese cervical spine CT images. The results indicated that the AH, MH, and PH of the C4-C7 were 3.95–4.29 mm, 5.22–5.72 mm, and 3.16–3.24 mm respectively. The results of Dong’s study were like those of this study, and the MH was greater than the AH and greater than the PH. These measurements are essential for the design of the implant height of the ATPRS intervertebral fusion system. Proper implant height after decompression and fusion surgery helps to distribute stress evenly between adjacent intervertebral discs and facet joints, thus maintaining normal range of motion (ROM) in the cervical spine [32]. Conversely, both excessively high and low implant heights can have adverse effects, such as increased stress or abnormal distribution at the implant-endplate interface, restricted flexion–extension movement of the cervical spine, cervical kyphosis, implant subsidence, and heterotopic ossification [33].

Morphologically, the upper endplate of the cervical vertebrae is generally flatter, while the lower endplate is typically more concave. Feng et al. [34] conducted a study on the anatomical morphology of cervical vertebral endplates in 138 cases and found that the depth of concavity in the upper endplate was greater than that in the lower endplate, with a depth of (1.88–2.13) mm for the upper endplate of C3-C7 and (0.62–0.84) mm for the lower endplate of C3-C7. In our study, the curvature diameter of the lower end plate of the cervical spine increased from C3 to C6 in both male and female groups, that is, the depth of concavity in the lower endplate decreased from C3 to C6. This can guide the design of the upper surface of the ATPRS intervertebral fusion system.

According to the measurement results, the screw entry area (P_1_P_2_) of the ATPRS intervertebral fusion system ranged from 2.92 to 6.08 mm in the male group and from 2.32 to 5.12 mm in the female group. The P_1_P_2_ showed an increasing trend from C3 to C7 in both male and female groups, which may be related to the changes in intervertebral space height and pedicle position. The average value of P_1_P_2_ in the male group was greater than that in the female group, aligning with the morphological and anatomical differences between male and female cervical spines. And there were statistical differences between them (*p* < 0.05). These results indicated that there is a screw placement area in the cervical intervertebral space, ensuring non-overlapping screw paths for the upper and lower screws. Furthermore, the screw placement area gradually increases from C3-C7 intervertebral space. The ATPRS intervertebral fusion system is feasible in morphology and anatomy.

Previous studies have demonstrated that the length, diameter, and thread type of the screw can impact its fixation strength [35–37]. Through the measured data, the screw length of the ATPRS intervertebral fusion system exceeded 20 mm, providing a greater length advantage compared to VBS and Zero-P [38]. Therefore, in theory, the ATPRS intervertebral fusion system can provide stronger stability for the injured cervical spine. Although the screw length of the ATPRS intervertebral fusion system is still shorter than that of ATPS technology, it can reduce the risk of damaging the nerve tissue. The measurement results showed that the length of the upper screw and lower screw increased from C3 to C7 in both men and women. Furthermore, the screw length of men was larger than that of women.

Tables 7 and 8 indicate that within the screw placement area of C3-C7, the upper screw inclination angles ranged from 47.73° to 66.76° for males, and 49.15° to 65.66° for females. Similarly, the lower screw inclination angles ranged from 48.05° to 65.35° for males, and 49.42° to 63.29° for females. This is larger than the inclination angle of the conventional Zero-P screw, which may have a certain impact on the insertion of the screw during operation [18]. Due to the size and location of the incision during clinical operations, it may pose challenges for the ATPRS intervertebral fusion system to place the screws to the pedicle root. Therefore, further design and improvement are needed in the future.

The screw cranial/caudal tilted angle was also different in different cervical segments. Through measurement, it was found that the screw cranial and caudal tilted angle ranged from 29.12° to 36.95° and from 32.06° to 39.56°, in the male group within the screw entry area of C3-C7; In the female group, the lower screw cranial angle ranged from 27.29 to 37.20° and the upper screw caudal tilted angle ranged from 30.97° to 38.92°. Overall, the cranial/caudal tilted angle of the ATPRS intervertebral fusion system exceeds that of the ATPS technology, but it remains within the angle range of the conventional Zero-P, indicating its feasibility [17, 39].

However, this study also has some limitations. First, the sample size is small, the elderly population was not included in the study, and the representativeness is limited, so further large sample measurement is needed. Second, this study is the preliminary study of the ATPRS intervertebral fusion system, and its biomechanical properties need to be verified by later experiments. Third, the operability of the ATPRS intervertebral fusion system needs to be further improved.

In conclusion, this study verified the feasibility of the ATPRS intervertebral fusion system in cervical morphology and anatomy through cervical CT imaging research and preliminarily obtained the relevant parameters of the ATPRS intervertebral fusion system, which provided some basis for the design of ATPRS intervertebral fusion system.

## Data Availability

Datasets are available from the corresponding author on a reasonable request.
